# Antioxidant and Anti-Atherogenic Activities of Essential Oils from *Myrtus communis* L. and *Laurus nobilis* L. in Rat

**DOI:** 10.3390/nu14071465

**Published:** 2022-03-31

**Authors:** Dyana Odeh, Nada Oršolić, Marija Berendika, Domagoj Đikić, Sandra Domjanić Drozdek, Sandra Balbino, Maja Repajić, Verica Dragović-Uzelac, Irena Landeka Jurčević

**Affiliations:** 1 Department of Animal Physiology, Faculty of Science, University of Zagreb, 10000 Zagreb, Croatia; dyana.odeh@biol.pmf.hr (D.O.); domagoj.djikic@biol.pmf.hr (D.Đ.); 2 Department of Food Quality Control, Faculty of Food Technology and Biotechnology, University of Zagreb, 10000 Zagreb, Croatia; mberendika@veinst.hr (M.B.); sandra.domjanic-drozdek@zvu.hr (S.D.D.); 3 Department of Food Engineering, Faculty of Food Technology and Biotechnology, University of Zagreb, 10000 Zagreb, Croatia; snedjer@pbf.hr (S.B.); maja.repajic@pbf.unizg.hr (M.R.); vdragov@pbf.hr (V.D.-U.); 4 Laboratory of Chemistry and Food Biochemistry, Faculty of Food Technology and Biotechnology, University of Zagreb, 10000 Zagreb, Croatia; ilandeka@pbf.hr

**Keywords:** myrtle and laurel essential oils, lipid parameters, atherogenic indicators, antioxidative activity in tissue, antiatherogenic activity

## Abstract

Essential oils (EOs) from aromatic and medicinal plants, such as myrtle (*Myrtus communis* L.) and Laurel (*Laurus nobilis* L.), are gaining popularity as a potential ingredient in functional foods and nutraceuticals. This study aims to investigate whether the essential oils (EOs) could be effective in weight control, antioxidative and antilipidemic status of rats by affecting microbiota and its enzymes activity and whether changes in intestinal enzyme activity affect the health of rats. The intragastric application of laurel and myrtle EOs to rats for two weeks affects weight loss, reduces glycolytic activity, lipid parameters (cholesterol, triglycerides, low-density lipoprotein cholesterol (LDL-C) and very low-density lipoprotein cholesterol (VLDL-C)) and atherogenic indicators, leading to cardiovascular protection. Laurel EO can be an excellent candidate for the treatment of drug-induced obesity and related diseases, since it affects lipid metabolism in the liver and inhibits the enzymes responsible for the metabolism of carbohydrates into glucose in the digestive tract, leading to weight loss. In contrast, myrtle EO shows a better antioxidant capacity in most tissues, except kidneys, where it causes a pro-oxidative effect, compared to laurel EO. Myrtle EO increases the permeability and instability of the erythrocyte membrane, resulting in a loss of selectivity for the entry of toxic substances into the cell. On the other hand, myrtle EO leads to intestinal inflammation by reducing the number of probiotic bacteria and increasing *Enterobacter*.

## 1. Introduction

Plant-derived essential oils (EOs), such as myrtle (*Myrtus communis* L.) and laurel (*Laurus nobilis* L.), are a complex of different lipophilic, low molecular weight, aromatic, and volatile compounds, usually consisting of monoterpenes, sesquiterpenes, phenylpropanes and their oxygenated derivatives, such as alcohols, aldehydes, esters, ketones, phenols, and oxides. Numerous in vitro and in vivo studies have shown that EOs from aromatic and medicinal plants exhibit different biological and pharmacological properties, e.g., antibacterial, antifungal, antiviral, anti-inflammatory, immunomodulatory, antioxidant, and wound healing effects, such as in sunburns, chemical burns, radiation burns, and eczema [1,2,3,4,5]. In folk medicine, plant extracts and EOs play an important role as therapeutic substitutes in the prevention and treatment of cardiovascular diseases due to their antiatherogenic, hypocholesterolemic, hypotensive, and anti-inflammatory properties [1,2,3]. Several studies have shown that Eos could affect weight gain and improve resistance to infection in swine and poultry [1] and that they have beneficial effects on antioxidant status and intestinal morphology and barrier in animals [4]. EOs have shown effectiveness in treating damaged mucous membranes of the gastrointestinal tract, including oral ulcers, gastric ulcers, and stress ulcers [5]. In addition, EO constituents, such as carvacrol and thymol, were able to modify the intestinal microbial composition of weaned piglets by increasing some beneficial bacterial species in order, such as *Bacillales*, *Lactobacillales*, and in the family *Streptococcaceae*, and *Veillonellaceae*, while in poultry they were able to increase the number of *Lactobacilli* in the intestine and reduce the amount of *E. coli* and *Clostridium perfringens* [6,7]. Appropriate gut microbiota structure and metabolite functions are essential for maintaining homeostasis, while gut dysbiosis contributes to atherosclerosis, hypertension, heart failure, arrhythmia, cardiac tumors, and others [8]. The advantage of EOs in pharmacological applications is their fat solubility, low molecular weight, and small size. Moreover, isolated EO compounds are able to pass through the skin, mucosa, and cell membranes and thus enter the systemic body circulation [9,10]. Due to their good applicability, EOs are widely used in cosmetics, medicine, and food industries to control both human and animal pathogens and as substitutes for dietary antibiotics in farm animal production [2,4,5,10]. Furthermore, EOs possessing insecticidal activity can be used in agricultural fields as biopesticides and can be highly effective on insecticide-resistant insects and replace the use of chemical pesticides as a green alternative.

To date, multiple experiments have been performed on myrtle and laurel EO action on bacteria in vitro, although there is a lack of knowledge on how EOs influence microbial communities in vivo under natural conditions as well as on their antioxidant capacity in tissues, antilipidogenic status and atherogenic indices.

One of the main objectives of this study is, therefore, to complete the insufficient information on the effect of EOs of aromatic and medicinal plants, such as myrtle and laurel, on the intestinal microbiota and their enzymatic activity in healthy rats as an possibility to prevent the development of potential diseases in hosts.

*Bifidobacteria*, along with *Lactobacilli*, are important Gram-positive, lactic acid producing bacteria that are most dominant in adulthood and represent an important part of the normal intestinal microbiota of various mammalian species, maintaining their physiological functions throughout their life, such as maintaining intestinal and epithelial integrity, harvesting energy, protecting against carcinogens and pathogens, and maintaining host immunity. The disruption of their composition leads to dysbiosis associated with a variety of pathological diseases [6,11]. In addition, *Enterobacteriaceae* is a large family of Gram-negative bacteria, including many of more familiar pathogens, such as *Salmonella*, *Escherichia*, *Klebsiella*, *Proteus*, *Enterobacter*, *Citrobacter*, *Yersinia*, *Hafnia*, *Serratia* and *Shigella* [12]. Enterobacter species are responsible for many nosocomial infections, and less commonly community-acquired infections, including urinary tract infections (UTI), respiratory infections, soft tissue infections, osteomyelitis, and endocarditis. Members of the gut microbiota in the human and animal large intestine exhibit a variety of enzymatic activities with a potential impact on their health through the biotransformation of secondary plant products and xenobiotic compounds. For example, β-glucuronidase is capable of releasing carcinogens and mutagens from hepatically derived glucuronic acid conjugates and cancer patients exhibit higher β-glucuronidase activities than healthy controls [13], while fecal β-glucuronidase activity in rodents is increased after the consumption of a high-protein/high-fat diet [14] and decreased after the consumption of diets high in carbohydrates. β-glucosidase may exhibit either toxic/mutagenic or health-promoting effects depending on the form of aglycones from different plant glucosides. β-galactosidase is also a glycoside hydrolase enzyme that catalyzes the hydrolysis of β-galactoside into monosaccharides by breaking of a glycosidic bond. This enzyme activates the final step in the process of carbohydrate digestion, which leads to the breakdown of disaccharides and oligosaccharides into absorbable glucose. Therefore, the inhibition of these hydrolytic enzymes may ameliorate postprandial hyperglycemia through restraining the influx of glucose from the intestinal tract into the blood vessels. Nevertheless, more scientific data are needed to confirm the various health claims about the effectiveness of the EOs, especially their effect in the prevention and treatment of cardiovascular diseases due to their antiatherogenic hypocholesterolemic, hypotensive, and anti-inflammatory properties.

In the present study, the following hypothesis was therefore set: the dietary EOs of aromatic and medicinal plants, such as myrtle and laurel, could be effective in the weight control and the antioxidative and antilipidemic status of rats by affecting the microbiota and its enzyme activity. Accordingly, these EOs and their active components may be promising therapeutic agents for cardiovascular disease.

The objective of this study is to investigate whether lactic-acid-producing bacteria, as most dominant bacteria included in the physiological, pathological and immunological process, can be changed after laurel and myrtle EO intake and whether these changes influence the body weight and the antioxidative and antilipidemic status of rats by affecting intestinal enzyme activity and their microbial abundance.

## 2. Material and Methods

### 2.1. Plant Materials

Dry leaves of bay laurel (*Laurus nobilis* L.) and myrtle (*Myrtus nobillis* L.) (Table 1), collected in November 2020, were procured from Šafram Ltd. (Zagreb, Croatia) and Suban Ltd. (Strmec Samoborski, Croatia), respectively, and stored at ambient temperature. Prior to analysis, the leaves of both plants were ground into a semi-fine powder using a commercial grinder (Waring WSG30, Sprzęt Laboratoryjny i Medyczny 358 Labpartner KBS, Warsaw, Poland). The obtained powders were analyzed for total solids by drying to constant mass at 105 °C [15] and the content of dry matter in the samples was >95%.

### 2.2. Extraction of the Essential Oil

Ground leaves (100 g) were subjected to hydrodistillation for 3 h in Clevenger-type apparatus. The obtained EO samples were dried over anhydrous sodium sulfate and stored in dark glass vials at −18 °C until further analysis.

### 2.3. Determination of the Composition of EOs

Prior to analysis, the EO samples were diluted (1:99) in *n*-hexane (HPLC purity, 95%) and internal standard was added (nerol, c = 10.650 mg/mL). The composition of EOs was determined using an Agilent Technologies 6890N Network GC System gas chromatograph (Santa Clara, CA, USA) equipped with an Agilent Technologies 5973 inert Mass Selective Detector (MS) (Santa Clara, CA, USA). Separation was performed on Agilent HP-5MS, 5%-phenyl-methylpolysiloxane capillary column (30.00 m × 0.25 mm × 0.25 μm) with helium used as the carrier gas at constant flow 1 mL/min. A sample (1 μL) was injected in the split ratio of 100:1. The following column temperature program was applied: initial temperature 60 °C, then 60–145 °C (3 °C/min), 145–250 °C (30 °C/min) and retention for 3 min at maximum temperature (250 °C). The total duration of the chromatographic analysis was 34.83 min. The temperature of the injector was 250 °C, while temperatures of MS transfer line, MS source and quadrupole were 280, 230 and 150 °C, respectively. The electron energy for the ionization of the sample molecules was 70 eV, and the mode of operation was set as selected ion monitoring (SIM). The parameters of the mass spectrometer were set to a reading speed of 1 reading s^−1^ (scan s^−1^), and the range of separation of mass and charge (*m*/*z*) in the range 30–550.

The identification of volatile compounds was performed by comparing the retention indexes and mass spectra (*m*/*z*) of the analyzed compounds with the retention indexes and mass spectra of commercial standards, and comparing the obtained mass spectra with those in the NIST database using Agilent G1701DA MSD ChemStation Data Analysis software (Agilent Technologies, Santa Clara, CA, USA). Additionally, to confirm the identified compounds, their retention index was calculated and compared with the data reported in the literature. In order to calculate the retention index of the isolated volatile compounds, a standard mixture of C_7_–C_30_ alkanes was prepared and analyzed under the same chromatographic conditions as the samples. Quantitative values for individual volatile components were calculated from the calibration equations of standard compounds where compounds lacking standards and were quantified based on the compound of the most similar chemical structure from the same chemical group. The analysis was performed in triplicate and the results are expressed in mg/mL oil as mean ± standard deviation.

### 2.4. Animal Study

#### 2.4.1. Experimental Animals and Ethics

All experimental procedures were carried out in the experimental laboratory of the Department of Animal Physiology, Faculty of Science, University of Zagreb, on adult female Sprague Dawley (220–240 g) rats, three months old, obtained from the Department of Animal Physiology, Faculty of Science, University of Zagreb. The experiment was conducted following the guidelines, such as the guidelines in force in Republic of Croatia (Law on the Welfare of Animals, NN135/06 and NN37/13), and the Guide for the Care and Use of Laboratory Animals, DHHS Publ. # (NIH) 86–123, National Research Council. The experiment involved 15 rats, which were divided into 3 groups: a control group and 2 experimental groups, laurel oil group and myrtle oil group. The animals were kept in conventional conditions in a 12:12 h light: dark cycle with constant conditions of room temperature (25 ± 1 °C), and air humidity of 50 ± 10% and housed in groups of five animals in plastic cages. The rats were fed with a commercial pelleted diet 4RF21 (Mucedola, Italy; Batch No. 238603, shape 12 mm), which included wheat, wheat straw, hazelnut skins, maize, soy bean dehulled, corn gluten feed, fishmeal, dicalcium phosphate, sodium chloride, whey powder, soybean oil, and yeast, and contained 12% moisture, 18.5% protein, 3% fats, 6% crude fibers, 7% crude ash, E672 (vitamin A), E671 (vitamin E), E1 (iron, Fe), E2 (iodine, I), E3 (cobalt, Co), E4 (copper, Cu), E5 (manganese, Mn), and E6 (zinc, Zn) [13,15]. Water was provided ad libitum. Ethical approval for the study was obtained from the ethical committee (Faculty of Science, University of Zagreb, Croatia; approval code: 251-58-10617-21-3, date of Approval: 20 September 2017). During housing, the health status of the animals was also monitored.

#### 2.4.2. Animals, Experimental Treatment and Organ Processing

After acclimatization, the rats (*n* = 15) were treated by the intragastric (*ig*) administration of laurel and myrtle EOs once daily for 14 days. All rats in experimental groups received the equivalent dose of 0.5 mL of myrtle or laurel EO, i.e., 1 µL per 1 g of weight. Laurel and myrtle EOs were mixed with sunflower oil (1:1) before administration. The control received 0.5 mL of sunflower oil. The dosing was adjusted according to the status of the rat’s weight on a daily basis. At the end of the experiment, on day 15 after treatment, rats were anesthetized using a mixture of ketamine (Narketan^®^10, Vetoquinol AG, Belp Bern, Switzerland) at dose of 75 mg/kg with xylazine (Xylapana^®^ Vetoquinol Biowet Sp., Gorzow, Poland) at dose of 10 mg/kg and exsanguinations was performed from axillary blood vessels for biochemical analysis. The blood was kept on ice and then centrifuged to obtain serum. The unhemolyzed serum was used for the analysis of total cholesterol (TC), triglyceride (TG), high-density lipoprotein (HDL-C) and low-density lipoprotein (LDL-C) concentrations. All lipid parameters were analyzed based on enzymatic methods as described by [15,16,17,18]. The liver, kidney and spleen of each rat were dissected and weighed, and tissues were used to determine oxidative and anti-oxidative status by measured the lipid peroxidation, glutathione levels, and catalase activity (CAT) as well as for antioxidative capacity analysis by methods, such as ferric reducing antioxidant power (FRAP) and 2,2′-azino-bis(3-ethylbenzothiazoline-6-sulfonic acid) (ABTS)^•+^ free radical scavenging activity. Fresh fecal samples (weight 0.1 g) were collected from each rat for the isolation and enumeration of probiotic bacteria (*Lactobacillaceae* and *Bifidobacteriaceae*) and *Enterobacteriaceae*, assessing microbiota enzyme activities (β-glucuronidase, β-glucosidase, β galactosidase activity, and pH value). To assess whether EOs affect animal weight, all animals were weighed during the experiment.

### 2.5. Body Weight

Animal body weight was monitored by weighing the animals during the experiment (on the first day of the experiment, every seven days during the experiment and on the day of sacrifice) using a digital scale (Kern KB 2000-2N, Balingen, Germany; d = 0.01–2000 g) and the percentage of body weight change was calculated according to the formula:

(1) $$\mathrm{Weight}\;\mathrm{change}\;\left(\%\right)=\frac{\left(\mathrm{final}\;\mathrm{weight}-\mathrm{initial}\;\mathrm{weight}\right)\times 100}{\mathrm{final}\;\mathrm{weight}}$$

### 2.6. Sample Collection, Preparation and Storage of Tissue Samples

Blood was sampled directly from the heart according to the guidelines of the Clinical Laboratory Standards Institute [19] and the World Health Organization [20]. Blood was collected in tubes without anticoagulants (serum) for biochemistry. All organs were iso-lated (liver, spleen, lungs, kidneys, brain, intestines and intestinal contents) and one part of the tissue was placed in Bouin’s solution for histological analysis, and the other part was frozen at −80 °C for further processing.

### 2.7. Relative Mass of Individual Organs

At the end of the experiment, the rat organs were isolated and weighed on a digital scale (Electronic balance ABS 220-4, Kern & Sohn, Balingen, Germany). The effect of laurel and myrtle EOs on the relative mass of each individual organ was calculated according to the formula:

(2) $$\mathrm{Relative}\;\mathrm{organ}\;\mathrm{mass}\;\left(\%\right)=\left(\frac{\mathrm{final}\;\mathrm{organ}\;\mathrm{mass}}{\mathrm{final}\;\mathrm{rat}\;\mathrm{mass}}\right)\times 100$$

### 2.8. Antioxidant Status of Tissues

Since the liver and kidney are key organs in direct contact with the drug, either for its antioxidant or toxic effect, the antioxidant capacity of the liver and kidney rat tissues was examined to test different antioxidant mechanisms using the following methods: (i) Ferric-reducing antioxidant power (FRAP) assay; (ii) ABTS assay; and (iii) oxidative stress markers, such as malondialdehyde (MDA), glutathione level (GSH), and catalase activity (CAT), which are described in [21,22]. Other tissues, brain, spleen and lungs were used only to assess markers of oxidative stress. Tissue supernatant samples were centrifuged at 20,000× *g* for 15 min at 4 °C and the supernatant was used for analysis following protocols described in our previous study [21]. 

The antioxidant capacity of liver and kidney after treatment with laurel and myrtle EOs was assessed by the FRAP and ABTS method described in detail in [21]. Briefly, the freshly prepared FRAP reagent (1.5 mL) was mixed with 200 µL of water and 50 µL of the tissue sample or blank standard sample with 50 µL water, and incubated for 4 min at room temperature. The absorbance was recorded at λ = 595 nm with a Libro S22 spectrophotometer (Biochrom Ltd., Cambridge, UK) and the ferric-reducing ability of the liver and kidney tissue homogenate was calculated according to the standard curve and expressed as nmol Fe^2+^ per mg of protein in samples.

In the ABTS assay, 20 μL of the tissue supernatant was mixed with 2 mL of an ABTS^•+^ solution (7 mM ABTS^•+^ solution with a freshly prepared 140 mM potassium peroxydisulfate solution mixed in equal proportions), and incubated for 6 min. After incubation, the absorbance was recorded at a 734 nm against a blank using a Libro S22 spectrophotometer (Biochrom Ltd., Cambridge, UK). The results of the liver and kidney samples are expressed as nmol Trolox equivalents per mg of protein in the tissue homogenate.

The malondialdehyde (MDA) level, biomarker for lipid peroxidation, was quantified in tissue homogenates by the method described in [21,22], following a reaction of thiobarbituric acid (TBA) with the formed MDA. After the incubation of tissue homogenates with TBA at 95 °C for 60 min, the pink reaction product TBARS (thiobarbituric acid reactive substance) was determined spectrophotometrically with a wavelength of 532 nm. Total MDA concentration was calculated using the extinction coefficient for MDA (ε = 1.56 × 10^5^/M cm). The concentration of lipid peroxides was expressed as nmol MDA/mg protein.

The total glutathione (GSH), intracellular and extracellular, protective antioxidant level (μmol/mg proteins) was measured at 412 nm wavelength using the spectrophotometric method by mixing 40 μL of 10 mM 5,5′-dithiobis-(2-nitrobenzoic acid) (DTNB, Ellman’s Reagent) with 20 μL of the tissue supernatant pretreated with 40 µL of 0.035 M HCl and incubated for 10 min. DTNB reacts with GSH to form chromospheres, 5-thionitrobenzoic acid (TNB) and GS-TNB.

The CAT activity was assayed by monitoring the H_2_O_2_ degradation, which was measured spectrophotometrically by the decrease in absorbance at 240 nm using the spectrophotometer UV-160 (Shimadzu, Kyoto, Japan) as described in [21,22]. The reaction mixture was prepared by mixing 10 mM H_2_O_2_ in 50 mM phosphate buffer, pH = 7.0. This reaction mixture (980 µL) was mixed with the supernatant of the tissue homogenate (20 µL) and the CAT activity was calculated using the molar absorption coefficient of 39.4/M cm for H_2_O_2_. The specific activity was expressed as U/mg protein.

The carbonyl groups were determined in liver and kidney tissues proteins, according to the Reznick and Packer method [23]. The method is based on the reaction of carbonyl groups of the protein chain with 2,4-dinitrophenylhydrazine (DNPH) in an acidic medium to form 2,4-dinitrophenylhydrazone. The absorbance measurement at 370 nm (Libra S22, Biochrom Ltd., Cambridge, UK) against a blank (2 M HCl). The concentration of carbonylated proteins was calculated using the molar extinction coefficient (ε = 0.011 µ/M) and expressed as nmol/mg protein according to the following formula:

(3) $$\mathrm{Wc}=\frac{\left(A_{\mathrm{sample}}-A_{\mathrm{blank}\;}\right)\;}{\varepsilon }$$

where Wc = carbonylated proteins, A = absorbance and ε = molar extinction coefficient.

### 2.9. Blood Sample Collection and Laboratory Analysis

#### 2.9.1. Estimation of Serum Liver Enzyme Activity and Kidney Function

The unhemolyzed serum was collected and frozen at −80 °C until the further processing of biochemical parameters. All biochemistry analyses were conducted according to the recommendations of the International Federation of Clinical Chemistry (IFCC) methods in enzymology [24] and were performed with commercial kits (Sigma-Aldrich, St. Louis, MO, USA) on the Hitachi 717 automatic analyzer (Hitachi, Tokyo, Japan), as described in [16]. The serum biochemical parameters for the renal and hepatic function included the concentration of alkaline phosphatase (ALP-U/L), alanine aminotransferase (ALT-U/L), aspartate aminotransferase (AST-U/L), amylase (AMY-U/L), urea (mmol/L), creatinine (CRE-µmol/L), glucose and total protein (TP-g/L) levels.

Glycemic changes (%) were calculated according to the formula:

(4) $$\mathrm{Glycemic}\;\mathrm{changes}\;\left(\%\right)=\frac{\left(G_{x\;}-G_{0\;}\right)}{G_{0}}100$$

where G_0_ = blood glucose level at 0 day and G_x_ = blood glucose level at 15th day.

#### 2.9.2. Lipid Parameters and Atherogenic Risk Predictor Indices (ARPI) Calculation

The unhemolyzed serum were used for the analysis of TC, TG, HDL-C and LDL-C concentrations. All lipid parameters were analyzed based on the enzymatic methods as described by [16,17,18].

Briefly, the serum levels of TG were measured using the glycerol-3-phosphate oxidase/phenol and aminophenazone method. TG was enzymatically hydrolyzed to glycerol and free fatty acids using specific lipases, which were subsequently oxidized into hydrogen peroxide (H_2_O_2_) using glycerol kinase and glycerol phosphatase. The hydrogen peroxide reacts with 4-aminophenone and 4-chlorophenol under peroxidase catalytic action into colored quinonimine as a red day.

The serum levels of the total cholesterol (TC) concentration were measured at 512 nm using the cholesterol oxidase/phenol and aminophenazone method. Briefly, cholesterol esters were cleaved through the action of cholesterol esterase producing free cholesterol and fatty acids. Cholesterol oxidase catalyzed the oxidation of cholesterol to cholest-4-en-3-one and hydrogen peroxide, which affects the oxidative coupling of phenol and 4-aminoantipyrine, forming a characteristic red dye.

The analysis of HDL-C involved a two-step procedure. Firstly, it involves the precipitation of the lipoprotein fractions of LDL-C and very low-density lipoprotein cholesterol (VLDL-C) in the samples using phosphotungstic acid and magnesium ions, while HDL-C remains in the supernatant. The second step involves HDL-C quantitation contained in the supernatant using the cholesterol (reagents) modified enzymatic method, as described in [16,17,18]. In the presence of peroxidation, the generated hydrogen peroxide reacts with 4-aminoantipyrine, forming a purple-bluish dye that is directly proportional to the cholesterol concentration. Direct LDL–C was measured using the Hitachi 717 automatic analyzer (Hitachi, Tokyo, Japan), while VLDL-C was estimated by Friedewald’s formula (VLDL-C = TG/5).

After determining the concentration in mmol/L of the TC, TG, HDL-C and LDL-C fractions, the atherogenic risk index (ARI), percentage protection, atherogenic risk predictor indices (ARPI), cardioprotective index (CPI), and the indicator of insulin resistance, as TG/HDL-C ratio were calculated using the following arithmetic formula, described in [17,18].

ARI or atherogenic coefficient (AC):

(5) $$\mathrm{ARI}=\frac{\left(\mathrm{TC}-\mathrm{HDL}-C\right)}{\mathrm{HDL}-C}$$

(6) $$\mathrm{Percentage}\;\mathrm{protection}=\frac{\left({\mathrm{ARI}}_{\mathrm{Negative}\;\mathrm{control}\;\mathrm{group}}-{\mathrm{ARI}}_{\mathrm{Treated}\;\mathrm{group}\;}\right)}{{\mathrm{ARI}}_{\mathrm{Negative}\;\mathrm{control}\;\mathrm{group}}}$$

where:

Negative control group = untreated group.

Treated group = laurel-and-myrtle-EO-treated rat’s groups.

ARPI-1 or AIP: $\mathrm{ARPI}-1=\log\frac{\mathrm{TG}}{\mathrm{HDL}-c}$

ARPI-2 = LDL-C/HDL-C

ARPI-3 or CRR: $\mathrm{ARPI}=\frac{\mathrm{TC}}{\mathrm{HDL}-C}$

CPI: $\mathrm{CPI}=\frac{\mathrm{HDL}-C}{\mathrm{LDL}-C}$

An indicator of IR: $\mathrm{IR}=\frac{\mathrm{TG}}{\mathrm{HDL}-C}$

A detailed description of the methods is presented in Oršolić, et al. [17]. ARI, atherogenic risk index; ARPI-1, 2, 3, atherogenic risk predictor index 1, 2, 3; AIP, atherogenic index of plasma; TG, triglyceride; HDL-C, high-density lipoprotein cholesterol; LDL-C, low-density lipoprotein cholesterol; CRR, cardiac risk ratio, TC, total cholesterol; CPI, cardioprotective index; IR, Insulin Resistance, EO, essential oil.

#### 2.9.3. Measurement of Erythrocyte Osmotic Fragility

The susceptibility of erythrocytes, i.e., red blood cells (RBC), to free radical oxidation generated in vivo was determined by measuring the amount of hemoglobin released from RBC based on the RBC resistance to lysis as a function of a decreasing NaCl concentration, as described in [17]. After the incubation of erythrocytes at different NaCl concentrations at room temperature and centrifugation, the optical density of the supernatant was determined spectrophotometrically at 540 nm using Spectrophotometer Libra S22 (Biochrom Ltd., Cambridge, UK). Erythrocyte osmotic fragility was expressed as % hemolysis as compared to the positive control group (100% hemolysis) in distilled water.

### 2.10. Intestinal Contents and Isolation of Gut Microorganisms

#### 2.10.1. Intestinal Content Sampling and Analysis of Gut Probiotic Bacteria and Enterobacteriaceae

Culture media with inhibitory activity on other microorganisms were used for the isolation and determination of probiotic bacteria and *Enterobacteriaceae*. It was prepared according to the manufacturer’s instructions. After sterilization, the media were cooled in a water bath to 47 to 50 °C and poured into sterile Petri dishes. De Man, Rogosa and Sharpe (MRS) agar, transgalctosylated oligiosaccharide-propionate (TOS) agar, violet-red bile glucose (VRBG) agar, Casein Soybean Digest Agar (CASO) agar and thioglycolate from Merck (Darmstadt, Germany) were used as culture media and broths for the isolation and enumeration of microorganisms.

According to the prescribed standard ISO methods, all microbiological analyses were performed. Following aseptic rules, 100 mg of colon content was sampled for the measurement of the glycolytic activity of intestinal microbiota enzymes, and for the isolation and enumeration of *Lactobacillaceae*, *Bifidobacteriaceae* and *Enterobacteriaceae* family on a specific culture medium. The intestinal contents were resuspended in a thioglycolate broth at a ratio of 1:10 (*w*/*v*) from which ten-fold serial dilutions were prepared up to 10^9^ for the isolation of probiotic bacteria and up to 10^6^ for the isolation of enterobacteria.

*Bifidobacteriaceae* was isolated and enumerated according to ISO 29981:2010 [25]. A total of 0.1 mL loopful cultured broth was inoculated on six TOS agar plates (dilutions from 10^7^ to 10^9^) and anaerobically incubated using Oxoid™ AnaeroGen™ 2.5 L Sachet (Thermo Fisher Scientific, Waltham, MA, USA) for 72 ± 2 h.

The isolation and enumeration of *Lactobacillaceae* was performed according to ISO 20128:2006 [26]. Similar to *Bifidobacteriaceae*, 0.1 mL loopful cultured broth was inoculated on six MRS agar plates (dilutions from 10^7^ to 10^9^) and microaerophilically incubated using Oxoid™ CampyGen™ 2.5 L Sachet (Thermo Fisher Scientific, Waltham, MA, USA) for 72 ± 2 h.

The detection and enumeration of *Enterobacteriaceae* was performed according to ISO 21528-2: 2017 [27]. Intestinal sample (1 mL) dilutions from 10^3^ to 10^5^ were inoculated into six sterile Petri dishes. Dissolved VRBG agar (approximately 10 mL) was added to each Petri dish and the closed plates were rotated to mix the inoculated contents. By hardening the agar, another 15 mL of VRBG agar was added and the plates were incubated at 37 ± 1 °C for 24 ± 2 h. After the growth of typical *Enterobacteriaceae* colonies, 5 colonies were isolated on CASO agar, whose incubation was performed for 24 ± 2 h at 37 ± 1 °C. The biochemical confirmation of the isolated colonies was made by oxidase assay (Merck, Darmstadt, Germany) and Gram staining was performed. *Enterobacteriaceae* are Gram-negative, oxidase-negative bacteria.

The total number of isolated microorganisms is expressed as the logarithm of the number of cells (log10 CFU/mL). The formula for calculating the number of cells is:

(7) $$\mathrm{CFU}=\left(\frac{\mathrm{number}\;\mathrm{of}\;\mathrm{colonies}\;\mathrm{grown}\;}{\mathrm{sample}\;\mathrm{volume}\;\mathrm{used}}\right)\times \;\mathrm{reciprocal}\;\mathrm{value}\;\mathrm{of}\;\mathrm{decimal}\;\mathrm{dilution}$$

where CFU = colony-forming unit.

#### 2.10.2. Fecal Enzyme Activity

β-galactosidase, β-glucuronidase and β-glucosidase enzyme activities were determined from intestinal contents. By the method of Juśkiewicz et al. [28], the bacterial glycolytic activity from intestinal contents was measured by the rate of liberating of *o*- and *p*-nitrophenols (PNP and ONP) from their nitrophenyl glucosides. The substrate *o*-nitrophenyl-β-d-galactopyranoside (5 mM) was used to determine the enzyme β-galactosidase, the substrate *p*-nitrophenyl-β-d-glucuronide (5 mM) was used for β-glucuronidase, and the substrate *p*-nitrophenyl-β-d-glucopyranoside (5 mM) was used for β-glucosidase (Sigma-Aldrich, St. Louis, MO, USA). The substrates were dissolved in 100 mL of 100 mM phosphate buffer (pH 7.0). The reaction mixture contained 0.2 mL of 1:10 (*v*/*v*) intestinal supernatant content, which was further diluted in 100 mM phosphate buffer and 0.3 mL of substrate solution, and the mixture was incubated for 10 min at 37 °C anaerobically. Upon the completion of incubation, 2.5 mL of cold sodium carbonate (0.25 M) was added to stop the reaction, and the absorbance of *p*-nitrophenol at λ = 400 nm and *o*-nitrophenol at λ = 420 nm was measured on a spectrophotometer (Libra S22, Biochrom Ltd., Cambridge, UK). The enzyme activity (U/g colon contents) was calculated according to the formula [14]:

(8) $$U=\frac{\left[\left(\frac{A}{a}\right)/10\right)/0.0182]}{139.11}$$

where U is enzyme activity, A is the absorbance; a is the slope of the direction (to determine the activity of the enzymes, β-glucuronidase and β-glucosidase are used from the calibration diagram for *p*-nitrophenol (PNP), and for the enzyme β-galactosidase from the calibration diagram for o-nitrophenol (OPN); 10 is the incubation time in minutes; 0.0182 is the amount of sample expressed in grams (in 0.2 mL of supernatant); and 139.11 is the Mr (PNP/ONP) in g/mol.

The test was performed in triplicates. The enzyme inhibitory rates of laurel and myrtle extracts were calculated according to the following equation:

(9) $$\mathrm{Inhibitory}\;\mathrm{rate}\;\left(\%\right)=\frac{\left(A_{\mathrm{control}}-{\;A}_{\mathrm{sample}}\right)}{A_{\mathrm{control}}}\times 100$$

where A = absorbance.

### 2.11. Histopathological Analysis of the Liver and Gut

The hematoxylin–eosin (H&E, Sigma, St. Louis, MO, USA) evaluation of the gut (ileum) and liver, and oil red lipid staining in the liver were performed. For H&E on ileum and liver samples, after washing with normal saline (NS), the intestinal samples were fixed in 4% paraformaldehyde overnight, transferred through the series of ethanol concentrations and embedded in paraffin. Thereafter, tissue slices (5 mm) were stained with hematoxylin and eosin (HE) and evaluated under a Nikon Eclipse E600 light microscope equipped with digital camera AxioCam ERc5s and ZEN2 lite software (Carl Zeiss Microscopy GmbH, Jena, Germany). For lipid staining with the oil red (Sigma, St. Louis, MO, USA) method, the liver slices were frozen, cut (15 µm sections) on the cryostat Leica CM 1850 (Leica Microsystems Nussloch GmbH, Nussloch, Germany) and stained with oil red stain for 7 min. After the oil red stain, the hematoxylin counterstain of the nuclei was applied to the sections for 3 min and washed 3 times in distilled water.

### 2.12. Statistics

The data were analyzed using the nonparametric Kruskal–Wallis test and were presented as mean ± standard error of the mean (SEM) values. The differences between the groups were made with multiple comparisons of mean ranks for all groups. Statistical analyses were performed using STATISTICA 13 software (StatSoft, Tulsa, OK, USA). The data were considered significant at *p* < 0.05

## 3. Results

### 3.1. Chemical Composition of EOs

**Figure 1:**
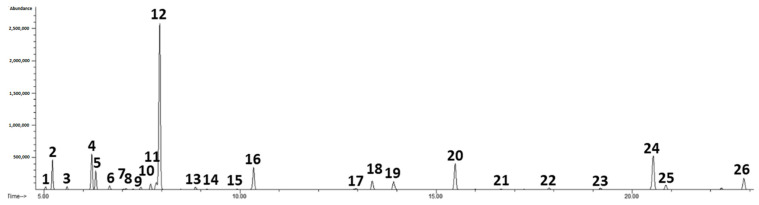
Chromatogram of volatile compounds of laurel essential oil (1 = α-thujene; 2 = α-pinene; 3 = camphene; 4 = sabinene; 5 = β-pinene; 6 = myrcene; 7 = α-phellandrene; 8 = 3-carene; 9 = α-terpinene; 10 = *p*-cymene; 11 = d-limonene; 12 = eucalyptol; 13 = γ-terpinene; 14 = *cis*-sabinene hydrate; 15 = α-terpinolene; 16 = linalool; 17 = δ-terpineol; 18 = terpinen-4-ol; 19 = α-terpineol; 20 = nerol (is); 21 = linalyl acetate; 22 = bornyl acetate; 23 = δ-terpinyl acetate; 24 = α-terpinyl acetate; 25 = eugenol; 26 = methyleugenol).

**Figure 2:**
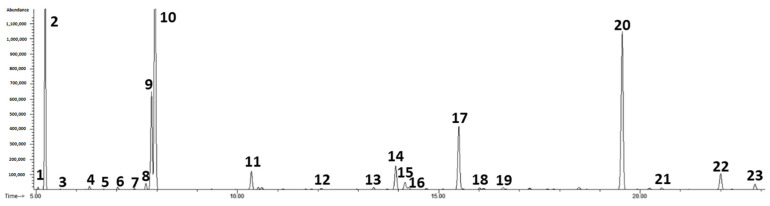
Chromatogram of volatile compounds of myrtle essential oil (1 = α-thujene; 2 = α-pinene; 3 = camphene; 4 = β-pinene; 5 = myrcene; 6 = α-phellandrene; 7 = 3-carene; 8 = *p*-cymene; 9 = d-limonene; 10 = eucalyptol; 11 = linalool; 12 = camphor; 13 = terpinen-4-ol; 14 = α-terpineol; 15 = myrtenol; 16 = estragole; 17 = nerol (is); 18 = carvone; 19 = geraniol; 20 = myrtenyl acetate; 21 = α-terpinyl acetate; 22 = geranyl acetate; 23 = methyleugenol).

The results revealed that the laurel EO contained a complex mixture of several components (Figure 1), predominately eucalyptol (347.45 ± 6.66 mg/mL), α-terpinyl acetate (148.48 ± 0.63 mg/mL), sabinene (62.85 ± 1.00 mg/mL), α-pinene (61.60 ± 0.90 mg/mL), linalool (55.44 ± 0.16 mg/mL), terpinen-4-ol (32.85 ± 0.22 mg/mL), methyleugenol (30.67 ± 0.23 mg/mL), β-pinene (28.18 ± 0.23 mg/mL), while other components were present in much lower concentrations (Table 2). Figure 2 shows a sample of the chromatographic profile of the volatile compounds of myrtle EO. The most abundant components found in the myrtle EO were eucalyptol (244.60 ± 1.63 mg/mL), α-pinene (193.75 ± 1.53 mg/mL), myrtenyl acetate (146.10 ± 0.84 mg/mL), d-limonene (69.25 ± 0.71 mg/mL), terpinen-4-ol (31.62 ± 0.20 mg/mL), α-terpineol (26.26 ± 0.34 mg/mL), geranyl acetate (20.71 ± 0.08 mg/mL) and others were found in less concentration (Table 2).

### 3.2. Body Weight Change

**Figure 3:**
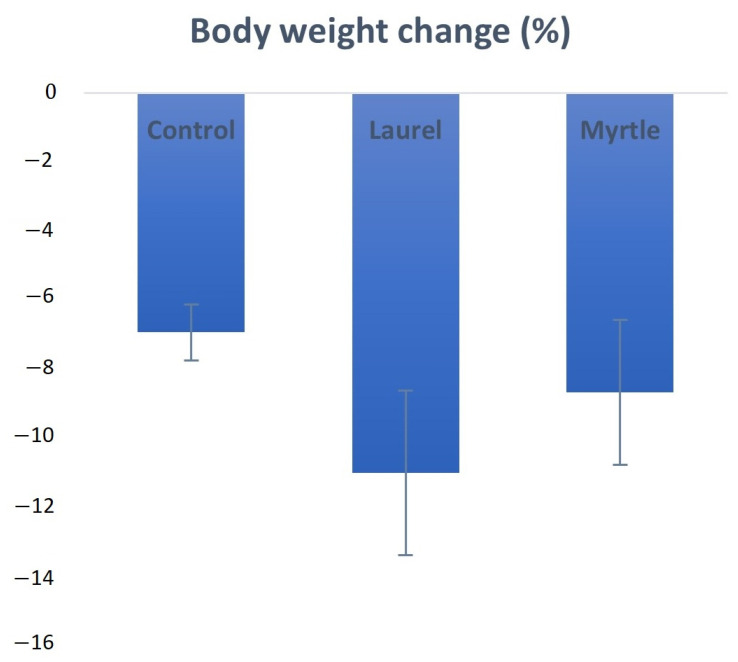
Evolution of the body weights of rats treated with laurel and myrtle EOs. Female rats (*n* = 5) were administered *ig* with laurel and myrtle EOs at a dose of 1 µL per 1 g of rats once daily for 14 days. Laurel and myrtle EOs were mixed with sunflower oil (1:1) and the total daily volume per rat was 0.5 mL. The control group was treated *ig* with 0.5 mL of sunflower oil. The results are expressed as the mean value of each experimental group ± SEM. Abbreviations: EOs, essential oils; *ig*, intragastric, SEM, standard error of the mean.

During the experimental period, no mortality was observed in the rats that were available for evaluation. The percentage of body weight change is shown in Figure 3, where a reduction in body weight is visible in the control as well as in the experimental groups, but without statistical significance. The highest percentage of change in the body weight of rats was caused by the application of laurel EO (−11.10%), followed by myrtle EO (−8.75%) and the control oil (−6.99).

### 3.3. Fecal Microbial Count

It is known that diet as well as a number of environmental factors and stress can affect the intestinal microbiome and have consequences on human and animal health. Thus, the consumption of high saturated and *trans*-fat diets is thought to increase the risk of cardiovascular disease through the up-regulation of blood total cholesterol and LDL-C [16]. On the other hand, health-promoting fats, such as mono- and polyunsaturated fats, are crucial in alleviating the risk of chronic disease. This study focused on changes in the total counts of *Lactobacillus*, *Bifidobacterium* and *Enterobacter* and on the specific enzymatic activity of the β-glucuronidase, β-glucosidase, and β-galactosidase derived from the intestinal flora of the rats after the application of myrtle and laurel EOs.

The lactic acid bacteria load was measured by plating on MRS agar, *Bifidobacterium* on TOS agar after anaerobic incubation at 37 °C for 72 h, while *Enterobacteriaceae* family by inoculating a sample on selective VRBG agar at 37 °C for 24 h.

**Figure 4:**
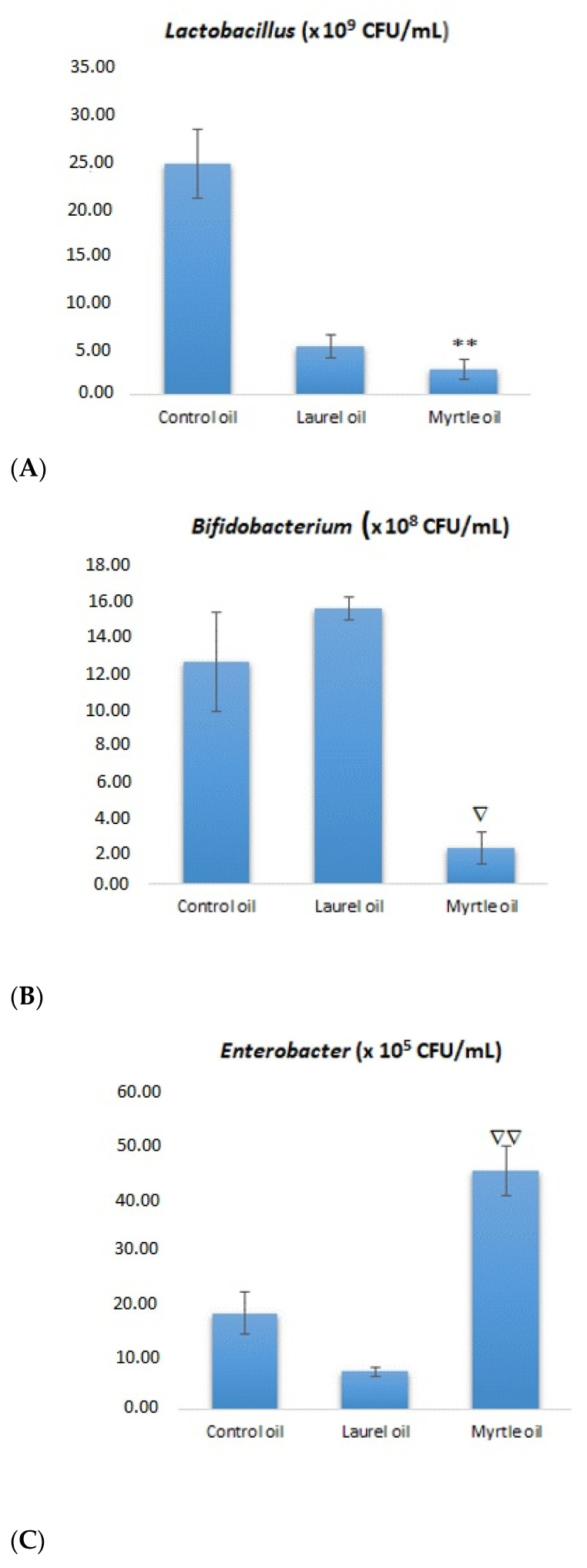
Effect of laurel and myrtle EO application on fecal microbial count of of *Lactobacillus* (**A**), *Bifidobacterium* (**B**) and *Enterobacteriaceae* (**C**). Female rats (*n* = 5) were administered *ig* laurel and myrtle EOs at a dose of 1 µL per 1 g of rats once daily for 14 days. Laurel and myrtle EOs were mixed with sunflower oil (1:1) and the total daily volume per rat was 0.5 mL. The control group was treated *ig* with 0.5 mL of sunflower oil. The results are expressed as the mean value of each experimental group ± SEM. * Significantly different in relation to the control group (** *p* < 0.01); ^∇^ Significantly different in relation to the laurel-EO-treated group (^∇^ *p* < 0.05; ^∇∇^ *p* < 0.01). Abbreviations: CFU, colony-forming unit.

Figure 4 depicts the fecal microbial count of rats after their treatment with EOs from laurel and myrtle. Interestingly, the number of *Lactobacillus* colonies was reduced in all experimental groups (laurel and myrtle) when compared to the control group as follows: 7.23 ± 1.26; 2.72 ± 1.11 (*p* < 0.01) vs. 24.88 ± 3.69 CFU × 10^9^/mL. However, the number of colonies of *Bifidobacterium* increased in laurel EO treatment rats, while it decreased in rats treated with myrtle EO, if compared with the control group; the number of CFU × 10^8^/mL was as follows: 15.85 ± 0.64, and 2.03 ± 0.92 vs. 12.78 ± 2.84. An unexpected situation was observed in the number of *Enterobacteriaceae* colonies; the treatment with myrtle EO induced a significantly higher CFU × 10^5^/mL in relation to laurel EO (*p* < 0.05) and control, but without significance. The two-week treatment with laurel and myrtle EOs had no influence on fecal pH when compared to the control (Table 3).

### 3.4. Fecal Bacterial Enzyme Activity

**Figure 5:**
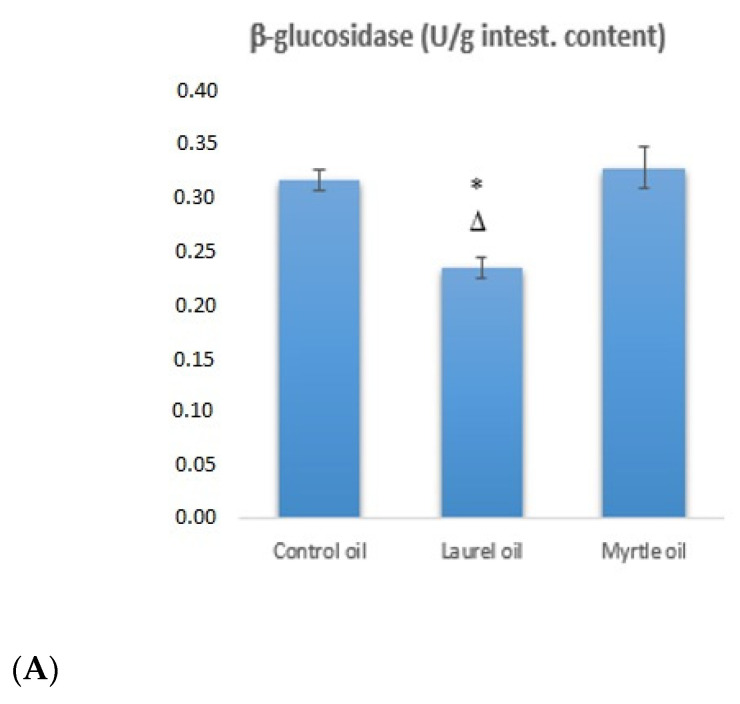

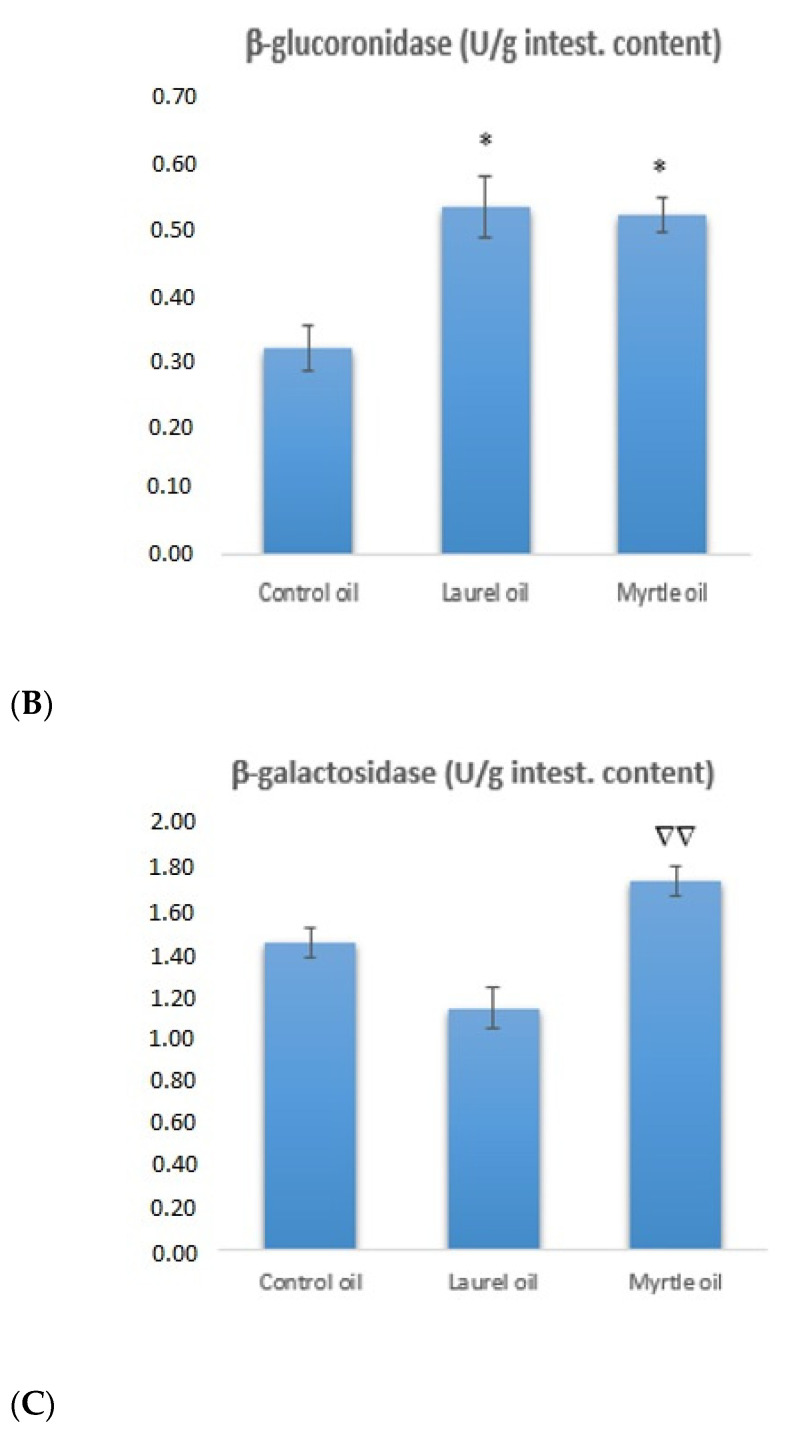
Activity of the bacterial enzymes β-glucosidase (**A**), β-glucuronidase (**B**) and β-galactosidase (**C**) in the colon contents of rats treated with laurel and myrtle EOs. Female rats (*n* = 5) were administered *ig* with laurel and myrtle EOs at a dose of 1 µL per 1 g of rats once daily for 14 days. Laurel and myrtle EOs were mixed with sunflower oil (1:1) and the total daily volume per rat was 0.5 mL. The control group was treated *ig* with 0.5 mL of sunflower oil. The results are expressed as the mean value of each experimental group ± SEM. * Significantly different in relation to the control group (* *p* < 0.05); ^∇^ Significantly different in relation to the laurel-EO-treated group (^∇∇^ *p* < 0.01). ^Δ^ Significantly different in relation to the myrtle-EO-treated group (^Δ^ *p* < 0.05). Abbreviations: intest., intestinal.

Freshly collected feces samples were examined for the enzymatic activity of the bacterial enzymes β-glucosidase, β-glucuronidase and β-galactosidase. Changes in the activity of bacterial enzymes are summarized in Figure 5. The application of laurel EO reduced the activity of β-glucosidase and β-galactosidase by 25.64 and 21.08%, but it increased the activity of β-glucuronidase by 68.46% (*p* < 0.05), as compared to the control group. The myrtle EO increased the enzymatic activity of β-glucosidase, β-glucuronidase and β-galactosidase by 3.87, 65.26 and 20.14% in relation to the control group.

### 3.5. Relative Organs Weights

**Figure 6:**
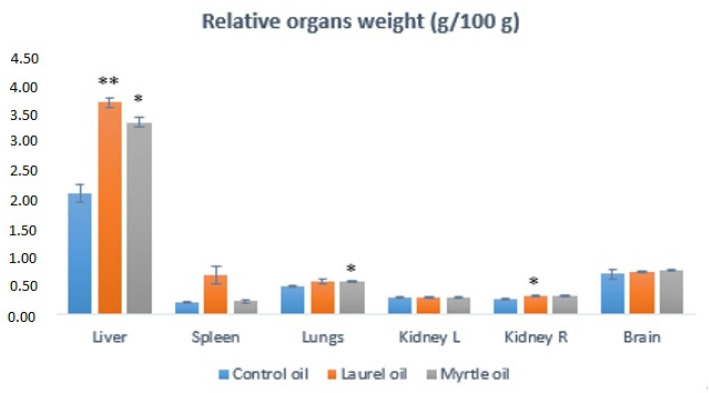
Effect of the laurel and myrtle EO application on relative organ weight. Female rats (*n* = 5) were administered *ig* with laurel and myrtle EOs at a dose of 1 µL per 1 g of rats once daily for 14 days. Laurel and myrtle EOs were mixed with sunflower oil (1:1) and the total daily volume per rat was 0.5 mL. The control group was treated *ig* with 0.5 mL of sunflower oil. The results are expressed as the mean value of each experimental group ± SEM. * Significantly different in relation to the control group (* *p* < 0.05; ** *p* < 0.01).

The relative weights of the liver of rats treated with laurel and myrtle EOs were increased significantly in relation to the control (*p* < 0.001; *p* < 0.05), while the other tissues were not affected by the dietary treatment, except the lungs in the rats treated with myrtle EO (*p* < 0.05) and the right kidney in the rats treated with laurel EO (Figure 6).

### 3.6. Antioxidative Capacity of the Liver and Kidney

**Figure 7:**
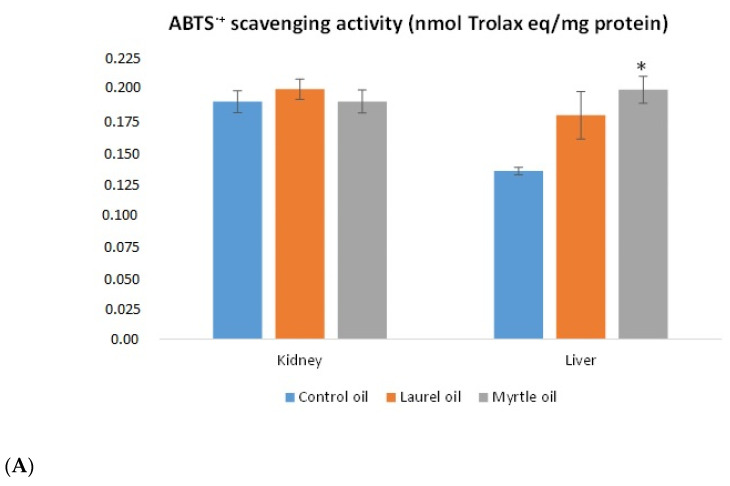

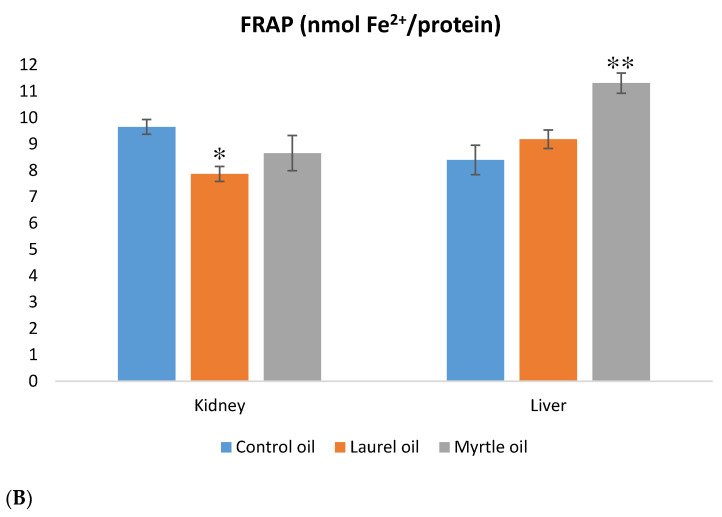
The effect of the laurel and myrtle EO application on the antioxidative capacity of the liver and kidney tissues homogenates measured by ABTS (**A**) and FRAP activity (**B**). Female rats (*n* = 5) were administered *ig* with laurel and myrtle EOs at a dose of 1 µL per 1 g of rats once daily for 14 days. Laurel and myrtle EOs were mixed with sunflower oil (1:1) and the total daily volume per rat was 0.5 mL. The control group was treated *ig* with 0.5 mL of sunflower oil. The results are expressed as the mean value of each experimental group ± SEM. * Significantly different in relation to the control group (* *p* < 0.05; ** *p* < 0.01). Abbreviations: ABTS, 2,2′-azino-bis(3-ethylbenzothiazoline-6-sulfonic acid); FRAP, ferric reducing antioxidant power; eq, equivalent.

The antioxidative capacity of the liver and kidney is shown in Figure 7. Unexpectedly, the treatment of rats with myrtle EO showed a significant increase in the liver antioxidant capacity when compared to the control, as confirmed by ABTS^•+^ scavenging activity (*p* < 0.05) and the FRAP (*p* < 0.01) method. The FRAP value was reduced in kidney tissue after the treatment with laurel EO compared to the control rats (*p* < 0.05).

**Figure 8:**
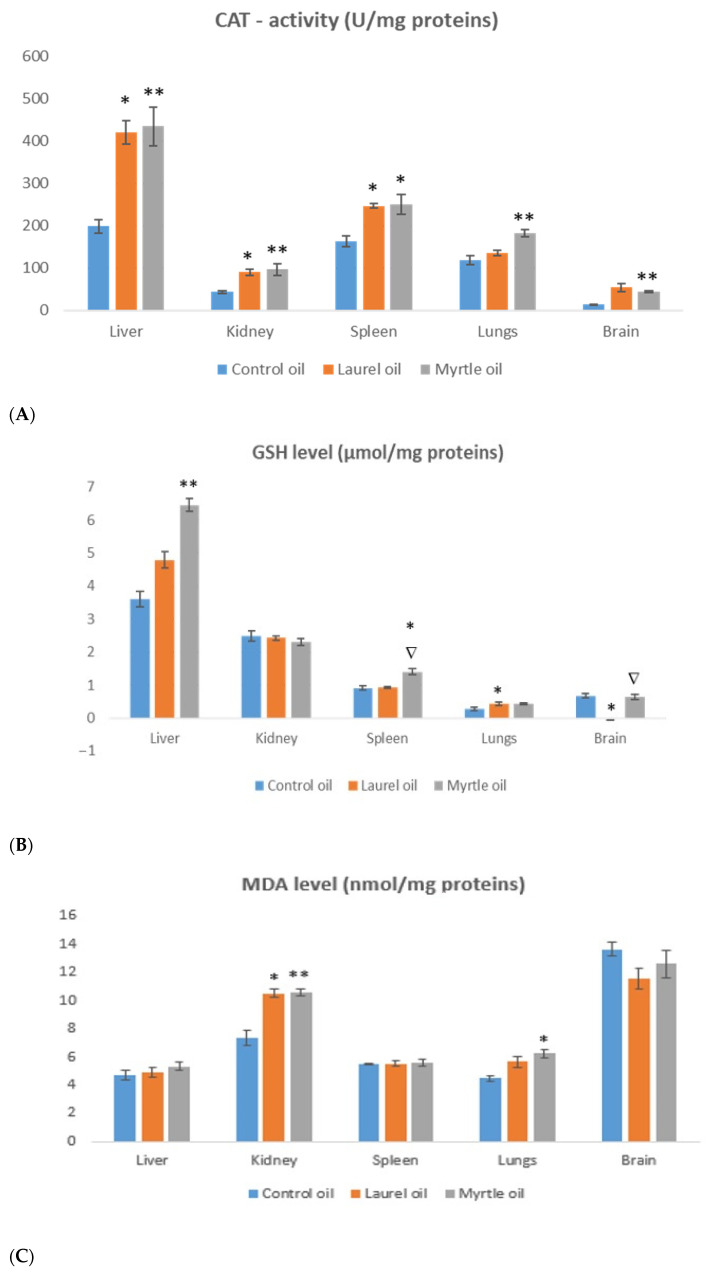

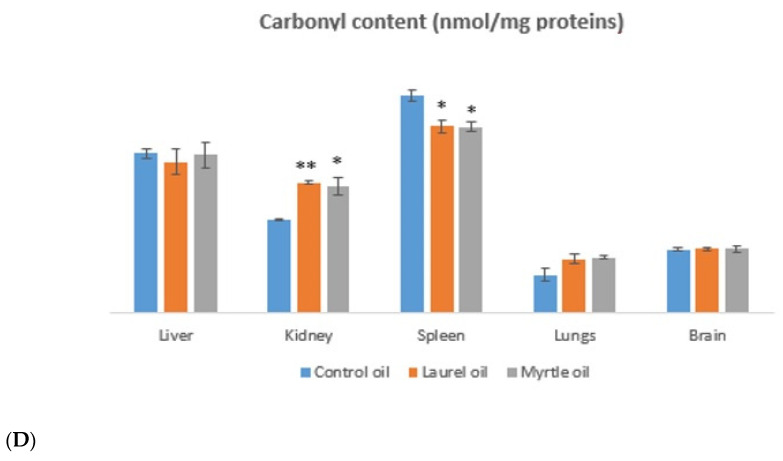
The effect of laurel and myrtle EO application on the oxidative stress biomarkers of the liver, kidney, spleen, lungs and brain tissues homogenates measured by the CAT activity (**A**), GSH (**B**) MDA (**C**) and carbonyl content (**D**). Female rats (*n* = 5) were administered *ig* with laurel and myrtle EOs at a dose of 1 µL per 1 g of rats once daily for 14 days. Laurel and myrtle EOs were mixed with sunflower oil (1:1) and the total daily volume per rat was 0.5 mL. The control group was treated *ig* with 0.5 mL of sunflower oil. The results are expressed as the mean value of each experimental group ± SEM. * Significantly different in relation to the control group (* *p* < 0.05; ** *p* < 0.01). ^∇^ Significantly different in relation to the laurel-EO-treated group (^∇^ *p* < 0.05). Abbreviations: CAT, catalase activity; GSH, total glutathione; MDA, malondialdehyde.

The treatment of rats with myrtle EO significantly increased the levels of MDA in the kidney (*p* < 0.01) and lungs (*p* < 0.05), GSH in the liver (*p* < 0.01) and spleen (*p* < 0.05) as well as CAT activity in the liver (*p* < 0.01), kidney (*p* < 0.01), spleen (*p* < 0.05) and lungs (*p* < 0.01) (Figure 8). The application of laurel EO in rats significantly increased the levels of MDA in the kidney (*p* < 0.05), CAT activity in the liver (*p* < 0.05), kidney (*p* < 0.05) and spleen (*p* < 0.05), when compared to the control. There is a significant difference in the GSH level in the spleen (*p* < 0.05) and brain (*p* < 0.05) between the treatment with myrtle and laurel EOs. The treatment of rats with laurel and myrtle EOs significantly increased the carbonyl content in the kidney (*p* < 0.01; *p* < 0.05) in relation to the control group, while the carbonyl content in the spleen was decreased (*p* < 0.05; *p* < 0.05) (Figure 8).

### 3.7. Osmotic Fragility Curve

**Figure 9:**
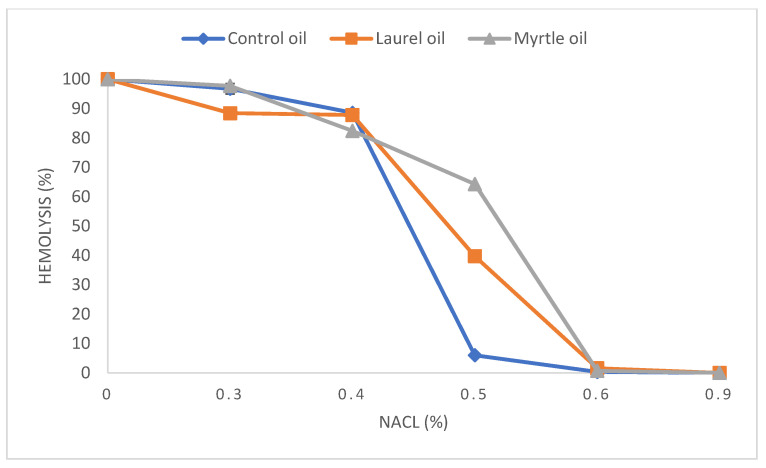
The effect of laurel and myrtle EO application on the osmotic fragility of blood samples at different concentrations of a sodium chloride solution. Female rats (*n* = 5) were administered *ig* with laurel and myrtle EOs at a dose of 1 µL per 1 g of rats once daily for 14 days. Laurel and myrtle EOs were mixed with sunflower oil (1:1) and the total daily volume per rat was 0.5 mL. The control group was treated *ig* with 0.5 mL of sunflower oil. The results are expressed as the mean value of each experimental group ± SEM.

The erythrocyte osmotic fragility test is used for the measurement of the erythrocyte membrane osmotic resistance. Specifically, the free radicals formed in vivo as well as the peroxidation of the unsaturated membrane lipid bonds increase the fragility and cell lysis of red blood cells. The EOs of laurel and myrtle possess antioxidant activity and are popular in the treatment of gastrointestinal diseases and anxiolytic symptoms, respectively [4,5,6,7]. Based on the above, it is important to assess whether laurel and myrtle EOs can protect erythrocytes from reactive oxygen species (ROS). Figure 9 shows the osmotic fragility curve of erythrocytes after the two-week treatment of rats with the EOs of laurel and myrtle. When comparing the treated and control groups, it can be observed that the 50% hemolysis of erythrocytes in the control group was at 0.45% of NaCl concentration, while in the laurel-EO-treated group, the 50% hemolysis of erythrocytes was at 0.49%. In the experimental group treated with myrtle EO, the 50% hemolysis of erythrocytes was at 0.55% NaCl. Statistical significance was present between the control and myrtle-EO-treated groups at 0.5% NaCl (*p* < 0.05).

### 3.8. Biochemical Parameters as Indicators of Liver and Kidney Function

Interestingly, most of the biochemical parameters were reduced or unchanged relative to the control group, except for ALP and amylase levels in the laurel-EO-treated rats (Table 4). ALT and ALP were significantly (*p* < 0.05) reduced in all experimental groups treated with EOs in comparison to control group as well as protein levels in the groups treated with laurel and myrtle EOs (*p* < 0.05).

### 3.9. Glycemic Change

**Figure 10:**
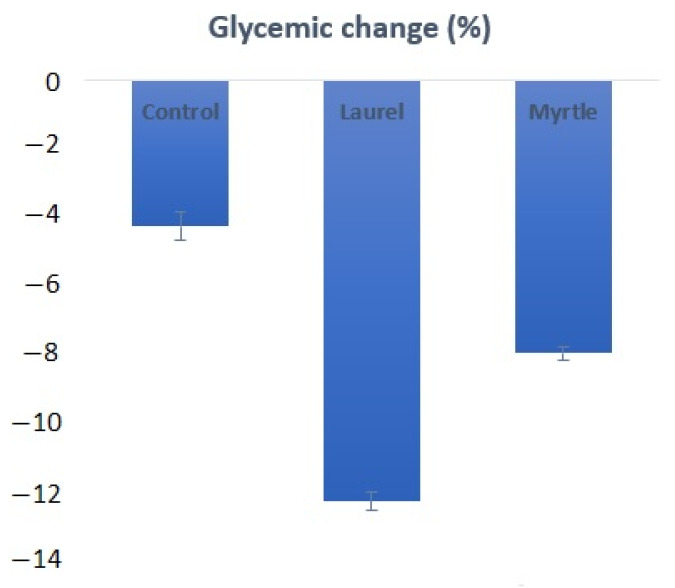
The effect of laurel and myrtle EO application on glycolytic change (%) in rat. Female rats (*n* = 5) were administered *ig* with laurel and myrtle EOs at a dose of 1 µL per 1 g of rats once daily for 14 days. Laurel and myrtle EOs were mixed with sunflower oil (1:1) and the total daily volume per rat was 0.5 mL. The control group was treated *ig* with 0.5 mL of sunflower oil. The results are expressed as the mean value of each experimental group ± SEM.

In order to determine the effect of laurel and myrtle EOs on the change in blood glucose levels, the animal’s blood sugar level was measured at the beginning of the experiment as well as at the end of the experiment. The initial value of the sugar level was 6.20 ± 0.53 mmol/L. At the end of the experiment, it was observed that the treatment of rats with the laurel EO led to the decrease in sugar levels by −12.419% and with the myrtle EO by −8.06%, while the control group had −4.30% (Figure 10).

### 3.10. Lipid Profile Analysis and Atherogenic Indices

Several studies have indicated that the type of fat, rather than the total amount of fat, in a diet plays a major role in hyperlipidemia [29]. Based on his, this study investigated and compared the lipid profile analysis and atherogenic indices of laurel and myrtle EOs on normocholesteremic rats. Table 5 shows the concentration of the lipid parameters of the experimental groups in which atherogenic indices were calculated using the appropriate formula as stated above.

The application of laurel and myrtle EOs to rats for 14 days led to a significant reduction (*p* < 0.05) in cholesterol levels by even 40 and 37% when compared to the control group. The triglyceride levels were reduced by about 60 and 55.70% after the application of the laurel and myrtle EOs. Interestingly, the HDL-C levels were slightly lower in the groups treated with EOs (~15%), while the LDL-C levels were reduced by 36.36 and 48.48% after the treatment with laurel and myrtle EOs, respectively.

The ARI and ARPIs are presented in Table 5. Specifically, Table 5 showed that laurel and myrtle EO application to rats exhibited the lowest ARI and ARPI-3, while the CPI index was increased when compared with the control rat group. In contrast, no effect of the laurel and myrtle EOs was observed on the TG/HDL-C ratio as a parameter of insulin resistance compared to the control group. When calculating the percentage of the atherogenic protection of the applied oils, laurel EO (40.68%) showed the best protective effect followed by myrtle EO (31.42%).

### 3.11. Morphometric Analyses of the Intestine and Reduction in Lipid Deposition in Liver

**Figure 11:**
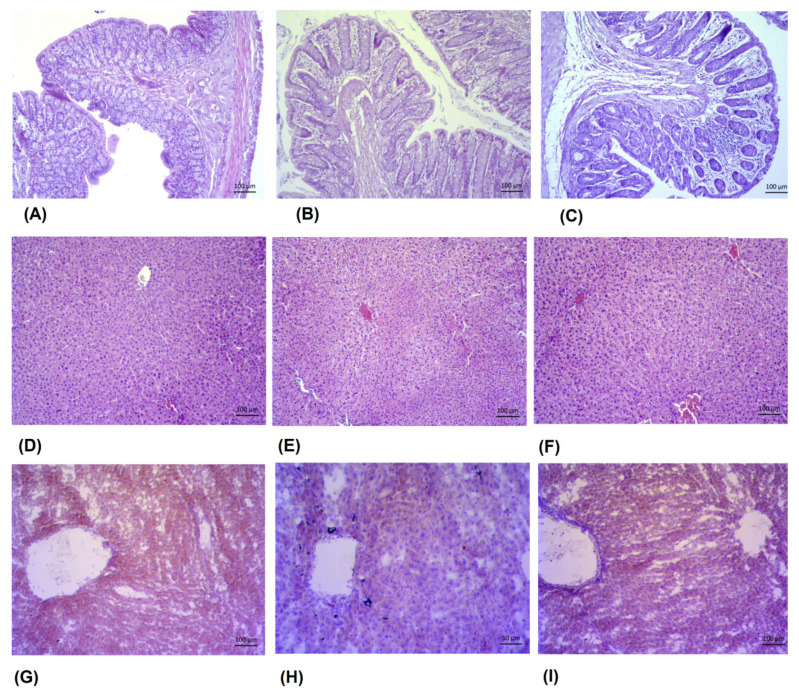
Histology of the gut (ileum) and liver of experimental animals. Hematoxylin–eosin (H&E) stain: (**A**) Control, ileum (H&E, 10×); (**B**) Laurel oil, ileum (H&E, 10×); (**C**) Myrtle oil, ileum (H&E, 10×); (**D**) Control, liver (H&E, 10×); (**E**) Laurel oil, liver (H&E, 10×); (**F**) Myrtle oil, liver (H&E, 10×). Lipid staining with oil red O stain and hematoxylin counterstain: (**G**) Control, liver (oil red O, 20×); (**H**) Laurel oil, liver (oil red O, 20×); (**I**) Myrtle oil, liver (oil red O, 20×).

Life in modern society, through numerous changes in lifestyle, eating habits and behavioral routines, leads to a significant increase in functional and immune disorders in the human gastrointestinal tract. Volatile essential oils from major food commodities, such as culinary and medicinal plants (spices), hold the potential to protect the digestive system from abusive agents and immune hypersensitivity by modulating inflammation and gut microbiome. Based on the above, and the changes observed in this paper, especially in the number of colonies of probiotic bacteria and *Enterobacteriaceae* and their enzymatic activity, as well as changes in lipid biochemical parameters and atherogenicity, we investigated the effects of laurel and myrtle EOs on structural and morphological changes in the liver and intestine (Figure 11).

In the intestine (ileum) of animals treated with both laurel oil (Figure 11B) and myrtle oil (Figure 11C), there were alterations in the mucosal epithelium and loss of structure of both villi and submucosa, compared to the control animals (Figure 11A). The laurel oil (Figure 11B) treatment caused a milder swelling of the mucosa, while the myrtle oil (Figure 11C) caused a pronounced swelling of the mucosa and the contraction of the villi length. Additionally, in the myrtle treatment (Figure 11C), more pronounced changes were present, where intestinal epithelial cell necrosis could be observed and the pronounced contraction of the villi and crypts were not preserved both in size and integrity, showing a progressive atrophy of the villi and dilation of the intestinal lumen, as well as detachment of the intestinal epithelia enlargement of lamina propria and submucosa (Figure 11C). Alterations in the Lieberkühn crypt glands were observed with the developed mucositis in the mice treated with myrtle oil (Figure 11C). Noted changes after myrtle oil treatment (Figure 11C) included cell inflammatory infiltrate, indicative of the inflammatory status. Within the intestinal crypts, the infiltration of immune cells, such as macrophages, neutrophils, eosinophils and lymphocytes, was observed. However, in the myrtle oil treatment (Figure 11C), there was still a partially preserved arrangement of the enterocytes and goblet cells, indicating early inflammation and mucositis with partially preserved absorptive function.

The liver structure was preserved in the treatments with both oil extracts (Figure 11 D–F). In general, the myrtle oil liver histology (Figure 11F) and lipid accumulation (Figure 11I) were not different from those of the control (Figure 11D,G). However, in the liver of animals treated with the laurel EO (Figure 11E), a slight vacuolization was observed, compared to the control animals (Figure 11D) and the myrtle EO-treated group (Figure 11F). The comparison of liver lipid staining with oil red (Figure 11G–I) revealed that the vacuolization in hepatocytes caused by the laurel oil (Figure 11H) was not lipophilic and that the observed change was hydropic vacuolization. Indeed, it seems that the treatment with the laurel oil caused a reduced lipid accumulation in the hepatocytes (Figure 11H) compared to the control animals (Figure 11G) and the myrtle EO-treated group (Figure 11I).

## 4. Discussion

Alterations to the microbiome caused by environmental changes in humans and animals (e.g., diet, antibiotics, xenobiotics, stress, viruses, bacteria, parasites, and age) can cause significant changes in the composition of the intestinal microflora [1]. In fact, it has been reported that dietary alterations are responsible for 57% of the gut microbiota’s entire variation, whereas genetic background may only contribute to 12% [30]. The disturbance of intestinal microflora (dysbiosis) may increase an individual’s susceptibility to infections and diseases, such as inflammation, cancer and cardiovascular diseases. Insufficient information on the effect of EOs on the intestinal microbiota and their enzymatic activity in healthy rats as an opportunity to prevent hosts from potential diseases was one of the objectives of this study.

By analyzing the data obtained in this study, it was observed that EOs, such as those extracted from myrtle and laurel, change the composition of the microbiota in the intestine (Figure 4). The changes in the number of colonies on selective media, such as *Lactobacillus*, *Bifidobacterium* and *Enterobacter*, and the changes associated with bacterial enzymatic activity were analyzed. According to the results, there seems to be a clear association between an increase in the number of *Enterobacter* colonies and the glycolytic enzyme activity (Figure 4 and Figure 5). After the treatment with myrtle EO, a 2.5-fold increase in *Enterobacter* colonies and a 6.3-fold decrease in *Bifidobacterium* were observed, when compared to the control. These changes in the increase in the number of *Enterobacter* and the strong decrease in *Bifidobacterium* colonies could be key factors for the increase in the bacterial enzymatic activity of β-glucosidase, β-glucuronidase and β-galactosidase. According to Little [31], glucuronidase activity may be regulated by the *Enterobacteriaceae* family of *Proteobacteria*, including *Escherichia*, *Salmonella*, *Klebsiella*, *Shigella*, and *Yersinia* pathobionts, which can cause inflammation and changes in the intestines, leading to alterations in the mucosal epithelium and the loss of the structure of both villi and submucosa (Figure 11C). It has been generally reported that Gram-negative bacteria are more resistant to myrtle extracts and essential oils than Gram-positive bacteria. This resistance is likely due to the fact that Gram-negative bacteria have a wall associated with an outer complex membrane, which slows down the passage of essential oil hydrophobic compounds [32].

On the other hand, the application of laurel EO to rats does not cause significant changes in the number of *Lactobacillus*, *Bifidobacterium* and *Enterobacter* colonies when compared to the control, but a reduced number of colony-forming unit (CFU) of *Lactobacillus* and *Enterobacter* as well as an increased number of CFU of *Bifidobacterium* were observed. It seems that an increase in the number of *Bifidobacterium* colonies may be associated with a decrease in the count of *Enterobacter* colonies [33] as well as a reduction in the activity of β-glucosidase and β-galactosidase by 25.64% and 21.08%, and an increase in β-glucuronidase activity by 68.46% as compared to the control group. However, it is possible that the decreased enzyme activity is the result of a two-fold reduction in the total number (*Lactobacillus*, *Bifidobacterium* and *Enterobacter*) of colonies compared to the control and myrtle EO experimental group. These data are consistent with other data [33] and our study (Table 2), which showed that the laurel EO contains compounds, such as eucalyptol (~350 mg/mL), α-terpinyl acetated, sabinene, α-pinene, linalool, terpinen-4-ol, methyleugenol, and β-pinene, which were identified as main ingredients against a wide range of microorganisms as well as being compounds with anti-parasitic activity [33]. In addition, these components show a strong anti-inflammatory activity and local anesthetic effect. Furthermore, eucalyptol (~250 mg/mL), α-pinene, myrtenyl acetate, d-limonene, terpinen-4-ol, α-terpineol and geranyl acetate were identified also as the main compounds in myrtle EO. The antimicrobial activities of myrtle EO were assayed against food-borne and clinical pathogens and food spoilage bacteria, including *Escherichia coli*, *Klebsiella aerogenes*, *Salmonella typhi*, *Pseudomonas aeruginosa*, *Proteus vulgaris*, *P. mirabilis*, *Campylobacter jejuni*, *Mycobacterium tuberculosis*, *Staphylococcus aureus*, *Micrococcus luteus*, *Streptococcus pneumoniae*, *S. pyogenes*, *S. agalactiae*, *Listeria monocytogenes*, *Bacillus subtilis* and some molds and yeasts [32]. The mechanism by which EOs act on microorganisms depends on their chemical composition and may include multiple modes of action. In some cases, it may be due to the hydrophobicity of the chemical (EO), which penetrates into the lipid bilayer of the cell membrane and makes the cells more permeable, leading to the leakage of vital cell contents. In addition, some compounds can cross the microbial cellular membrane. The interactions with membrane enzymes and proteins would cause an opposite flow of protons, affecting cellular activity, including energy production (membrane-coupled), membrane transport, and other metabolic regulatory functions, the synthesis of DNA and RNA, and destroy protein translation [34,35]. They can also degrade the cell wall and cytoplasmic membranes, cause the leakage of cellular components, and change fatty acid and phospholipid constituents.

The reduction in the number of *Enterobacter* colonies after the treatment with the laurel EO (Figure 4) should be noted as it may be an important strategy in human and animals, since high fecal *Enterobacter* levels are associated with inflammatory bowel disease and immune imbalance and exacerbate the inflammatory status of the gut epithelium [36]. Thus, among other components, α- and β-pinene and sabinene are known to possess anti-inflammatory activity in experimental models of inflammation [9,32]. It seems that a strategy to reduce *Enterobacter*-induced inflammation may be associated with the antioxidant activity of the laurel EO and its beneficial effect on intestinal microorganisms, such as *Bifidobacterium* spp., which could reduce the count of pathogenic bacteria, such as *Escherichia coli* and *Clostridium* spp., in the intestinal contents [36,37,38]. Thus, the increase in *Bifidobacterium* spp., in addition to reducing pathogenic bacteria, also reduces the harmful effect of enzymes, such as β-glucuronidase, β-glucosidase and β-galactosidase. Furthermore, a significantly higher β-glucuronidase activity in animals who had lost weight was noted (Figure 3). This observation agrees with a recent study reporting the increase in β-glucuronidase activity in obese volunteers following a weight loss diet [36,37]. On the other hand, the inhibition of β-galactosidase by the laurel EO may reduce the release of glucose and monosaccharides from carbohydrates, which play a potential role in controlling blood sugar levels, bringing about anti-hyperglycemia (Figure 5, Table 5). According to the results in this study, the inhibition of β-galactosidase by the laurel EO may be an important approach to reduce postprandial hyperglycemia by retarding glucose uptake through the inhibition of carbohydrate-hydrolyzing enzymes, such as β-galactosidase. The weight loss of rats after the laurel and myrtle EO treatments may be related to changes in their glycolytic activity, lipid parameter (cholesterol, triglyceride, LDL-C and VLDL-C) levels, atherogenic indicators and the percentage of atherogenic protection (Table 5 and Figure 10). The increased number of *Bifidobacterium* colonies in the intestines contributes to the antiatherogenic properties of the laurel EO as well as the protection of the liver and intestines (Table 5, Figure 11E,H). It has been confirmed that *Bifidobacterium* and *Lactobacillus* are responsible for the scavenging of hydroxyl radicals and superoxide anions and for producing antioxidants, such as glutathione transferase, CAT, superoxide dismutase (SOD), GSH and metal-chelating and antioxidant molecules, which could protect the intestine, liver and vascular system. Additionally, an additional explanation may be that probiotics can inhibit intestinal pathogens and reduce postprandial lipids, which are involved in oxidative damage (Table 5, Figure 11E,H). This increase in *Bifidobacterium* and reduction in lipid parameters and glucose also appear to be crucial for the greater reduction in rat body weight after the application of the laurel EO to rats (Figure 3). A similar effect on body weight was described with citrus peel EO and lime EO [39,40]. These data appear to support the traditional application of laurel EO to the digestive system in the treatment of digestive symptoms, such as epigastric bloating, indigestion, eructation, and bloating.

The data available to date on EOs are very different. For instance, according to Thapa et al. [41], EOs were shown to affect the cell integrity of Gram-positive bacteria, but had no effect on the loss of cell integrity and growth inhibition of Gram-negative bacteria [41]. Comparable in vivo studies also found inhibiting effects against pathogens, such as *C. perfringens*, *E. coli* or *Eimeria* species [42]. In contrast, Horošová et al. [43] reported that the same EOs, such as oregano EO, exhibited a strong bactericidal effect against Lactobacilli isolated from the fecal samples of chickens fed diets with oregano, indicating the potential negative effects induced by EOs on healthy intestinal bacteria. The negative effect on *Lactobacillus* in this study was particularly confirmed with the myrtle EO (Figure 4). Similar data on beneficial bacteria were shown by Thapa et al. [41], which showed the increased susceptibility of beneficial commensal *Faecalibacterium rausnitzii* to EOs rather than the pathogens. Furthermore, other research reported that EOs had no effect on the microbial population and composition in the digestive tract or fecal excretions of broilers [44,45].

Changes in fecal enzyme activities in response to diet have been mainly investigated for β-glucosidase and β-glucuronidase as possible biomarkers for colorectal cancer risk, breast, cervical, colon, lung and renal carcinoma and leukemia because of their critical role in the cleavage of xenobiotic compounds, carcinogenic metabolites and conjugated hormones that, once free, can re-enter the human body via the enterohepatic circulation. In addition to β-glucuronidases, involved in increasing hormone-dependent pathological changes and tumor formation, other studies suggest a hormone-related protection of colorectal cancer via the enzymatic activation of phytoestrogens and other compounds found in fish oil, cruciferous vegetables, and estrogens [46]. β-glucuronidases can be categorized according to structural-functional groups as: (i) β-glucuronidases of opportunistic bacteria as the major contributors to xenobiotic-induced toxicity in the gut; (ii) commensal bacteria, such as *Lactobacillus* spp. and *Bifidobacterium* spp., for maintaining a healthy level of gut bacterial β-glucuronidases. The latter group is essential for the recycling of important endogenous molecules and for the regeneration of beneficial natural products [47,48,49]. According to our data, it seems that the increase in β-glucuronidase activity with the laurel EO may be related to commensal bacteria, such as *Lactobacillus* spp. and *Bifidobacterium* spp., that maintain a healthy level of the gut, rather than high-risk for colorectal cancer [50], while the β-glucosidase activity did not show a strong relationship with high-risk diets [51]. On the contrary, it has been shown that the natural β-galactosidase inhibitors from the dietary plants with minimal side effects may be an effective strategy for alleviating postprandial hyperglycemia and for the treatment of type 2 diabetes. The activity of β-glucosidase not only contributes to the hydrolysis of glucose monomers from non-starch polysaccharides (e.g., cellulose and β-glucans), but it may also participate in the formation of toxic aglycons from plant glucosides [52]. According to the literature data [12,53,54], β-glucosidases can exert either beneficial or harmful effects, as they form aglycones from a range of different plant glucosides, which might exhibit either toxic/mutagenic or health-promoting effects [55]. Furthermore, β-glucosidases, on the other hand, seem to have a role in the bioavailability of plant polyphenols and the extraction of energy from insoluble fibers and other indigestible carbohydrates [36].

The antimicrobial activity of EOs shown in this study and also their antioxidant properties have been confirmed by many authors [2,4,5,6,7,42,43,44]. However, there are no data on their antioxidant capacity in tissues. Although in vitro assays are used for rapid screening, their results cannot be directly extrapolated to in vivo conditions. The rats as models are therefore used to simulate in vivo conditions for the evaluation of the antioxidant capacity of the used EOs. The antioxidant capacity of the liver and kidney rat tissues was confirmed by FRAP and ABTS free radical scavenging assays and by markers of tissue oxidative stress defense systems, such as MDA, GSH and CAT activity. The treatment with myrtle showed the best antioxidant capacity of the liver as indicated by the increased values obtained by the ABTS and FRAP analysis. In addition, an increase in the GSH and CAT activity levels was observed in almost all organs, except the GSH levels in the kidney, in which increased levels of MDA and carbonylated proteins were observed. The application of the laurel EO to rats also significantly increased the levels of MDA in the kidney and the CAT activity in the liver, kidney and spleen, when compared to the control. According to Kondratyuk and Pezzuto [56], the pH of biological tissues could also influence the antioxidative/pro-oxidative activity of phenolics compounds. In general, a decrease in pH increased iron-reducing activity and reduced the ability of phenolics to chelate and inhibit the catalytic activity of iron. Increasing pH increased deoxyribose and DNA oxidation. According to Harassi et al. [57] and Bouzabata et al. [58], the high concentration in oxygenated and hydrocarbon monoterpenes, methyl eugenol, eugenol, and, α-terpineol present in EOs or the absence of phenolic compounds might be responsible for the weak antioxidative activity in tissues. Thus, eugenol has been shown to have antioxidant activity in low concentrations, but it acts as a pro-oxidant in high concentrations. Monoterpene derivatives are the main compounds found in EOs, such as α-pinene, 1,8-cineole, linalool and linalyl acetate, while several hydrocarbons and oxygenated monoterpenes were present in smaller quantities, namely, limonene, α-terpineol, and geranyl acetate. According to the authors of [57,58], the weak antioxidant activity of methyl eugenol refers to the delocalized electron(s) or proton(s) of methyl eugenol.

Several health benefits have been suggested by the use of natural products or plant-derived EOs, while other studies have evaluated their health risks, so the knowledge of toxicity is crucial to evaluate risks/benefits. RBC hemolysis has long been used to measure free radical damage and counteraction by antioxidants and could be used for screening oxidizing or antioxidizing agents. RBCs are the primary targets of free radicals, owing to their high membrane concentrations of polyunsaturated fatty acids (linoleic and arachidonic acids, in particular) and O_2_ transport associated with redox active hemoglobin molecules, which are potent promoters of ROS. Oxidation depletes the membrane protein content, deforms RBCs, and disturbs the microcirculation by compromising blood flow and oxygen uptake and release. Ionic Fe^2+^ acts as a catalyst in redox reactions and lipid peroxidation and forms malondialdehyde (MDA) as the end product [17]. Using the osmotic fragility test of RBCs, the stability and functionality of the membrane after the treatment of rats with EOs were checked and the percentage of the lysis of RBCs in different concentrations of NaCl (0–0.9%) was analyzed. The application of myrtle EO to rats also showed significant changes in erythrocyte fragility. When comparing rats treated with myrtle EO and the control group, the myrtle EO-treated group had 50% hemolysis at 0.55% NaCl, while the 50% hemolysis of erythrocytes in the control group was at 0.45% of NaCl concentration. The laurel-EO-treated group had the 50% hemolysis of erythrocytes at 0.49% NaCl. It seems that the increase in the curve of osmotic fragility observed in the present study (Figure 9) may be related to the interaction of EOs in the regulation and diffusion across the membrane of erythrocytes. The explanation behind this observation lies in the properties of the lipid bilayer as the main constituent of cellular membrane, which plays an important function in membrane transport by facilitating the diffusion of liposoluble substances intracellularly. Thus, after the treatment with EOs, liposoluble substances, such as the derivatives of terpenes, easily pass across the membrane and alter cellular homeostasis. According to these findings, the EOs in our study can interfere with amphipathic membrane substances, causing changes in cell structure and disrupting cell diffusion and thus can be used for the penetration of the drug into the membranes or enhancers increased drug diffusivity through the membranes. However, when blood samples were incubated with isotonic saline, no disturbances in erythrocyte lysis were observed in relation to the control groups. It is evident that the interaction of the EOs with erythrocytes causes a decrease in the phospholipids and results in the instability of the erythrocyte membrane, which is not a positive physiological effect, but it leads to a decrease in membrane fluidity [59]. Such changes affect changes in permeability, altering intracellular flow and resulting in a loss of selectivity for the entry of toxic substances into the cell. It is possible that these changes may be associated with a small increase in the relative weight of the liver, kidneys, lungs and spleen as organs that are well supplied with erythrocytes and in the toxic effect of myrtle EO in the intestine together with an increased number of *Enterobacter*. The loss of selectivity of the intestinal barrier allows the easier penetration of toxic microbial components together with other toxic food components, leading to the disruption of epithelial integrity and the emergence of inflammatory processes.

## 5. Conclusions

In conclusion, the use of EOs in rats can alter the intestinal microflora and their enzymatic activity as well as induce antioxidative activity in the liver, while in the kidney, it may induce prooxidative effect. The treatment of rats with laurel and myrtle EOs affects weight loss, reduces glycolytic activity, lipid parameters (cholesterol, triglycerides, LDL-C and VLDL-C) and atherogenic indicators, leading to cardiovascular protection. The findings of the present study also suggest that laurel EO can be an excellent candidate for the treatment of drug-induced obesity and related diseases, since it affects the lipid metabolism in the liver and inhibits the enzymes responsible for the metabolism of carbohydrates into glucose in the digestive tract, leading to weight loss. In contrast, myrtle EO shows a better antioxidant capacity in most tissues, except the kidneys, where it causes a pro-oxidative effect, compared to laurel EO, as confirmed by FRAP analysis, ABTS^•+^ scavenging activity, content of GSH, CAT activity, MDA level and protein carbonylation. In addition, the interaction of myrtle EO with the erythrocyte membrane increases the instability and permeability of erythrocytes, resulting in the loss of selectivity for the entry of toxic substances into the cell. On the other hand, it reduces probiotic bacteria and increases *Enterobacter*, leading to intestinal inflammation. Due to the inflammatory and toxic effects of myrtle EO, more toxicological studies are needed, including dose-dependent exposure and chronic exposure studies, but also combinations with different components to ensure that myrtle EO is safe for human and animal health as well as the environment.

Furthermore, we believe and expect that the same beneficial effect of EOs will be applicable to humans.

## Figures and Tables

**Table 1:** List of essential oils (EOs) used in this study.

Essential Oil	Latin Name	Plant Family	Plant Tissues Used
Laurel oil	*Laurus nobilis* L.	*Lauraceae*	Leaves
Myrtle oil	*Myrtus communis* L.	*Myrtaceae*	Leaves

**Table 2:** Content and composition of volatile compounds in laurel and myrtle essential oil *.

Compound	Content in Laurel Essential Oil (mg/mL Oil)	Content in Myrtle Essential Oil (mg/mL Oil)
α-thujene ^a^	4.46 ± 0.07	0.013 ± 0.002
α-pinene	61.60 ± 0.90	193.75 ± 1.53
Camphene	11.08 ± 0.27	1.08 ± 0.08
Sabinene ^b^	62.85 ± 1.00	n.d.
β-pinene	28.18 ± 0.23	2.35 ± 0.03
Myrcene	16.60 ± 0.12	2.68 ± 0.01
α-phellandrene	3.12 ± 0.03	1.66 ± 0.01
3-carene	1.37 ± 0.01	0.48 ± 0.01
α-terpinene	14.04 ± 0.18	n.d.
*p*-cymene	7.03 ± 0.01	3.45 ± 0.01
d-limonene	10.61 ± 0.21	69.25 ± 0.71
Eucalyptol	347.45 ± 6.66	244.60 ± 1.63
γ-terpinene	5.25 ± 0.02	n.d.
*cis*-sabinene hydrate ^b^	0.87 ± 0.01	n.d.
α-terpinolene ^c^	4.17 ± 0.32	n.d.
Linalool	55.44 ± 0.16	19.36 ± 0.08
δ-terpineol ^d^	6.95 ± 0.05	n.d.
Terpinen-4-ol ^d^	32.85 ± 0.22	31.62 ± 0.20
α-terpineol	23.08 ± 0.08	26.26 ± 0.34
Linalyl acetate ^e^	4.42 ± 0.08	n.d.
Bornyl acetate ^d^	8.90 ± 0.03	n.d.
δ-terpinyl acetate ^d^	8.31 ± 0.11	n.d.
α-terpinyl acetate ^d^	148.48 ± 0.63	4.53 ± 0.05
Eugenol	14.34 ± 0.17	n.d.
Methyleugenol ^f^	30.67 ± 0.23	9.88 ± 0.07
Camphor	n.d.	0.56 ± 0.08
Carvone	n.d.	2.14 ± 0.07
Geraniol	n.d.	6.21 ± 0.04
Myrtenyl acetate	n.d.	146.10 ± 0.84
Estragole	n.d.	0.013 ± 0.003
Geranyl acetate ^g^	n.d.	20.71 ± 0.08
Myrtenol	n.d.	3.92 ± 0.04

* Data expressed as mean ± SEM (*n* = 3). ^a^ expressed on α-pinene; ^b^ expressed on 3-carene; ^c^ expressed on α-terpinene; ^d^ expressed on α-terpineol; ^e^ expressed on linalool; ^f^ expressed on eugenol; ^g^ expressed on geraniol. Abbreviations: SEM, standard error of the mean; n.d., not detected.

**Table 3:** The effect of laurel and myrtle EOs on the pH value of the intestinal contents in rats.

Treatments ^a^	pH Value (Mean ± SE)	Minimum	Maximum
Control	7.37 ± 0.17	6.84	7.7
Laurel	7.35 ± 0.21	6.82	7.96
Myrtle	7.44 ± 0.13	6.95	7.71

^a^ Female rats (*n* = 5) were administered *ig* laurel and myrtle EOs at a dose of 1 µL per 1 g of rats once daily for 14 days. Laurel and myrtle EOs were mixed with sunflower oil (1:1) and the total daily volume per rat was 0.5 mL. The control group was treated *ig* with 0.5 mL of sunflower oil. The results are expressed as the mean value of each experimental group ± SEM. Abbreviations: EOs, essential oils; SE, standard error, SEM, standard error of the mean.

**Table 4:** Effect of laurel and myrtle EO application on the biochemical parameters in rats.

Parameters	Treatments ^a^ (X + SEM)		
	Control	Laurel	Myrtle
ALP (U/L)	44.33 ± 3.60	68.00 ± 4.94 *^∆^	45.000 ± 3.596
ALT (U/L)	44.00 ± 3.48	27.33 ± 1.87 *	28.000 ± 1.317 *
AST (U/L)	92.67 ± 4.90	63.00 ± 4.31 *	61.333 ± 4.638 *
Amylase (U/L)	883.67 ± 43.66	1106.33 ± 63.99	993.667 ± 33.107
TP (g/L)	66.33 ± 0.56	61.67 ± 1.11 *	60.333 ± 0.211 **
GLU (mmol/L)	5.93 ± 0.40	5.43 ± 0.27	5.700 ± 0.179
UREA (mmol/L)	4.93 ± 0.24	5.67 ± 0.21	4.433 ± 0.220 ^∇∇^
Creatinine (µmol/L)	39.67 ± 4.89	30.67 ± 0.92	31.000 ± 0.365

^a^ Female rats (*n* = 5) were administered *ig* with laurel and myrtle EOs at a dose of 1 µL per 1 g of rats once daily for 14 days. Laurel and myrtle EOs were mixed with sunflower oil (1:1) and the total daily volume per rat was 0.5 mL. The control group was treated *ig* with 0.5 mL of sunflower oil. The results are expressed as the mean value of each experimental group ± SEM. * Statistically significant compared to the control group (* *p* < 0.05; ** *p* < 0.01). ^∆^ Statistically significant compared to the myrtle-EO-treated group (^∆^ *p* < 0.05); ^∇^ Significantly different in relation to the laurel-EO-treated group (^∇∇^ *p* < 0.01). Abbreviations: ALP, alkaline phosphatase; ALT, alanine aminotransferase; AST, aspartate aminotransferase; TP, total proteins, GLU, glucose; *ig*, intragastric; X, mean value.

**Table 5:** Effect of laurel and myrtle EO application on the serum lipid biochemical parameters, atherogenicity and atherogenic risk predictor indices in rats.

Parameters	Treatments ^a^ (X + SEM)		
	Control	Laurel	Myrtle
TC (mmol/L)	2.33 ± 0.15	1.40 ± 0.04 *	1.47 ± 0.13 *
TG (mmol/L)	1.17 ± 0.20	0.93 ± 0.09	1.03 ± 0.10
HDL-C (mmol/L)	0.68 ± 0.04	0.57 ± 0.02	0.55 ± 0.06
LDL-C (mmol/L)	0.11 ± 0.01	0.07 ± 0.02 *	0.06 ± 0.00 *
VLDL-C (mmol/L)	0.23 ± 0.04	0.18 ± 0.02	0.21 ± 0.02
ARI (AC) = ((TC-HDL-C)/HDL-C)	2.43 ± 0.13	0.19 ± 0.02 ***^∆∆^	1.67 ± 0.04 *
ARPI-1 (AIP) = (log (TG/HDL-C))	0.23 ± 0.04	0.212 ± 0.04	0.27 ± 0.04
ARPI-2 = (LDL-C/HDL-C)	0.16 ± 0.03	0.122 ± 0.00	0.10 ± 0.00
ARPI-3 (CRR) = (TC/HDL-C)	3.43 ± 0.13	2.441 ± 0.09 *	2.67 ± 0.16 *
CPI = HDL-C/LDL-C	6.18 ± 0.50	8.190 ± 0.34 *	9.71 ± 0.43 *
IR = TG/HDL-C	1.72 ± 0.15	1.63 ± 0.07	1.88 ± 0.13

^a^ Female rats (*n* = 5) were administered *ig* with laurel and myrtle EOs at a dose of 1 µL per 1 g of rats once daily for 14 days. Laurel and myrtle EOs were mixed with sunflower oil (1:1) and the total daily volume per rat was 0.5 mL. The control group was treated *ig* with 0.5 mL of sunflower oil. The results are expressed as the mean value of each experimental group ± SEM. * Statistically significant compared to the control group (* *p* < 0.05; *** *p* < 0.001). ^∆^ Statistically significant compared to the myrtle-EO-treated group (^∆∆^ *p* < 0.01); Abbreviations: TC, total cholesterol; TG, total triglyceride; HDL-C, high-density lipoprotein cholesterol; LDL-C, low-density lipoprotein cholesterol; VLDL-C, very-low-density lipoprotein cholesterol; ARI, atherogenic risk index; AC, atherogenic coefficient; ARPI-1, 2, 3, atherogenic risk predictor index 1, 2, 3; AIP, atherogenic index of plasma; CRR, cardiac risk ratio; IR, insulin resistance. ARPI-2 = (LDL-C/HDL-C) ratio > 2.3 is atherogenic and undesirable; ARPI-3 (CRR) = (TC/HDL-C) ratio > 3.33 is atherogenic and undesirable.

## Data Availability

The original contributions generated for this study are included in the article; further inquiries can be directed to the corresponding author.

## Sample Availability

Samples of the compounds are available from the authors.
